# Synthesis and Characterization of a Silica-Based Drug Delivery System for Spinal Cord Injury Therapy

**DOI:** 10.1007/s40820-019-0252-6

**Published:** 2019-03-11

**Authors:** Guodong Sun, Shenghui Zeng, Xu Liu, Haishan Shi, Renwen Zhang, Baocheng Wang, Changren Zhou, Tao Yu

**Affiliations:** 1 College of Chemistry and Material Sciences, Jinan University, Guangzhou, 510632 People’s Republic of China; 2 Department of Orthopedics, First Affiliated Hospital, Jinan University, Guangzhou, 510632 People’s Republic of China; 3 College of Traditional Chinese Medicine, Jinan University, Guangzhou, 510632 People’s Republic of China; 4 Tsinghua-Berkeley Shenzhen Institute, Tsinghua University, Shenzhen, 518055 People’s Republic of China

**Keywords:** Silica, Drug delivery, Spinal cord injury, Arctigenin, Astrocytes

## Abstract

Acute inflammation is a central component in the progression of spinal cord injury (SCI). Anti-inflammatory drugs used in the clinic are often administered systemically at high doses, which can paradoxically increase inflammation and result in drug toxicity. A cluster-like mesoporous silica/arctigenin/CAQK composite (MSN-FC@ARC-G) drug delivery system was designed to avoid systemic side effects of high-dose therapy by enabling site-specific drug delivery to the spinal cord. In this nanosystem, mesoporous silica was modified with the FITC fluorescent molecule and CAQK peptides that target brain injury and SCI sites. The size of the nanocarrier was kept at approximately 100 nm to enable penetration of the blood–brain barrier. Arctigenin, a Chinese herbal medicine, was loaded into the nanosystem to reduce inflammation. The in vivo results showed that MSN-FC@ARC-G could attenuate inflammation at the injury site. Behavior and morphology experiments suggested that MSN-FC@ARC-G could diminish local microenvironment damage, especially reducing the expression of interleukin-17 (IL-17) and IL-17-related inflammatory factors, inhibiting the activation of astrocytes, thus protecting neurons and accelerating the recovery of SCI. Our study demonstrated that this novel, silica-based drug delivery system has promising potential for clinical application in SCI therapy.

**Electronic supplementary material:**

The online version of this article (10.1007/s40820-019-0252-6) contains supplementary material, which is available to authorized users.

## Highlights

- With good biocompatibility, a silica-based drug delivery system was prepared and used effectively in vivo to prolong the duration of drug treatment.
- The prepared system can target spinal cord injury directly. Additionally, due to its small size (approximately 100 nm), it can penetrate the blood-spinal cord barrier.
- This system reduced the expression of interleukin-17 (IL-17) and IL-17-related inflammatory factors and can protect neurons and promote the recovery of spinal cord injury.

**Electronic supplementary material:**

The online version of this article (10.1007/s40820-019-0252-6) contains supplementary material, which is available to authorized users.

## Graphical abstract

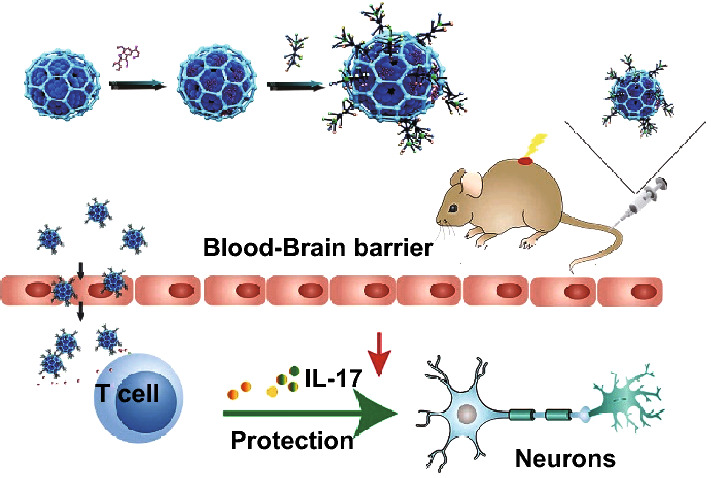

## Introduction

Spinal cord injury (SCI), a devastating condition, is becoming a serious health problem worldwide [1]. As a central pathological process of SCI, the inflammatory response plays a vital role in the clinical prognosis of SCI [2]. Therefore, curtailing this inflammation by modulating activated immunocytes (macrophages, astrocytes, etc.) has been suggested as a promising strategy [3, 4]; however, treatment of inflammation after SCI is limited [5]. Currently, methylprednisolone (MP) is most commonly used in SCI therapy [6], but MP has shown side effects, including increased risk of general and respiratory infections and hyperglycemia [7]. Therefore, it is crucial to find a safer and more effective drug for the treatment of SCI.

Arctigenin (ARC-G) is a lignan isolated from Chinese herbal medicine, *Arctium lappa* (Niubang). It has been in clinical use in China as a therapeutic agent to treat inflammatory conditions such as antipyretic colds and swelling of the throat [8]. It has also been reported that ARC-G has potential antitumor, antiviral, neuron-protective, and anti-inflammatory properties [9, 10]. The anti-inflammatory abilities of ARC-G are related to the suppression of pro-inflammatory cytokines. Recently, several researchers have reported that cytokines such as tumor necrosis factor-α (TNF-α), interleukin-17 (IL-17), IL-6, and IL-33 may be potential targets for the treatment of SCI [11–13]. IL-17 induces myelin destruction, neuronal death, and astrocyte toxicity in various inflammatory conditions of the central nervous system, such as multiple sclerosis (MS), experimental autoimmune encephalomyelitis (EAE), and SCI [14, 15]. Therefore, we hypothesized that downregulating IL-17 may be a promising strategy for the treatment of SCI.

ARC-G was reported to reduce IL-17 in the central nervous system of experimental autoimmune encephalomyelitis (EAE) mice [14]. Previous experiments by our group showed that application of ARC-G reduced the inflammatory response after SCI and promoted motor function [16]. However, the poor water solubility of ARC-G limited its clinical application because a high dose of the drug is needed to treat the disease. The anti-inflammatory drug treatment has, therefore, generated considerable controversy because high doses of such drugs induce deleterious side effects [17]. Thus, a challenge for SCI treatment is to search for an alternative delivery system for ARC-G that effectively controls inflammation at the site of SCI.

With the development of nanotechnology, biocompatible nanomaterials closely fulfill the requirement in the recovery of tissue engineering [18]. In photoacoustic molecular imaging applications, MoS_2_ nanosheets were supposed to be a suitable candidate due to their high loading capability and high optical absorption characteristics [19]. Functional graphene nanomaterials (FGNs) have been attracting more and more attention as an emerging platform in nanomedicine for drug/gene delivery, phototherapy, and bioimaging with excellent interaction and adhesive properties for protein, mammalian cells, and microbials [20]. A novel nanomaterial based on carbon nanotube-Fe_3_O_4_ was prepared to sensitively and reversibly trap, inactivate, and detach bacteria [21]. The authors believe that such nanoagents will not only have potential applications in pathogenic bacteria prevention but also provide a new pathway for wound disinfection, implant sterilization, and also live bacteria transportation. Photo-responsive liposomes with gold nanoparticles [22] and gold nanorod@polyacrylic acid/calcium phosphate (AuNR@PAA/CaP) yolk–shell nanoparticles [23] have been designed for controlled drug release in response to a change in the microenvironment. There is an increasing demand for drug delivery systems (DDS) that allow for controlled and triggered release upon stimulation. For the above systems, certain bottlenecks need to be overcome. The foremost concern is the stability of the system; the clearance of the delivery system from the human body and long-term effects of chronic usage have yet to be studied.

**Scheme 1:**
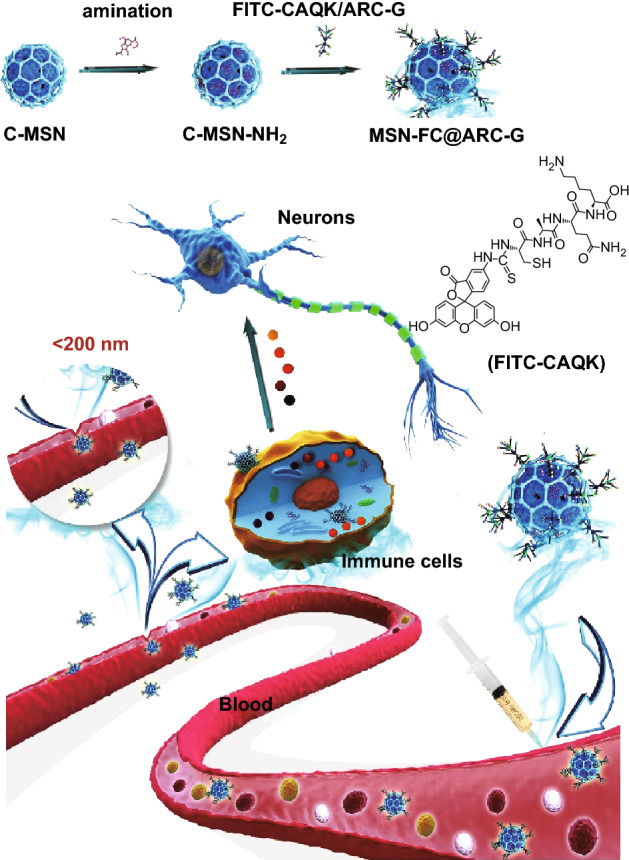
A system of the drug ARC-G-loaded MSNs with FITC-CAQK was designed to target SCI, regulate the activation of astrocytes, and protect nerves by reducing IL-17. The nanoparticle drug system loaded with ARC-G could improve the local cellular microenvironment

In the past decade, several studies have reported the application of nanoparticles for treatment of SCI [12, 24–27]. Compared with other nanoparticles, mesoporous silica nanoparticles (MSNs) have shown significant benefits as a drug delivery system over traditional drug nanocarriers due to their tailored mesoporous structure and high surface area [28, 29]. MSNs also provide a more advantageous choice for controlled and localized drug delivery due to their extensive nanopore structure in mesoporous silica [30–32]. Since anti-IL-17 has a positive role in functional recovery after SCI, we developed a cluster-like mesoporous silica/ARC-G (MSN/ARC-G) composite drug delivery system for the systemic treatment of mice with spinal cord injury through tail vein injection (Scheme 1).

The blood–spinal cord barrier (BSCB) is considered a major impediment to systemic treatment of SCI. To circumvent the BSCB, localized delivery of drugs within the spine has been explored, but this has limitations in clinical settings. In SCI, the BSCB is transiently disrupted allowing extravascular access for macromolecules and neuroprotective drugs from the systemic circulation. However, the lack of specific binding of passively accumulating proteins in the injured area can result in low retention and subsequent washout over time, resulting in significantly limited therapeutic efficacy of systemically administered drugs. Recently, in vivo phage display screening identified the peptide CAQK, which specifically targets brain and nerve injury sites [33, 34]. In our study, we grafted FITC-CAQK onto the surface of MSN/ARC-G (MSN-FC@ARC-G) to target the injured spinal cord tissue. We also conducted in vivo experiments to evaluate the effect of anti-IL-17 on the inflammation of the injury region and to study functional recovery after SCI.

## Experimental Section

### Preparation of Cluster-Like Mesoporous Silica Powder

Cluster-like mesoporous silica (C-MSN) was synthesized using a soft-templating method [35]. A mixture of 1.90 g of cetyltrimethylammonium tosylate (CTATos, MERK) and 0.35 g of triethanolamine (TEAH_3_) in 0.1 L of deionized water was stirred at 80 °C for 1 h; then, 14.58 g of tetraethyl orthosilicate (TEOS) was quickly added into the surfactant solution. The mixture was stirred at 80 °C with a stirring speed of 1200 rpm for another 2 h. The synthesized C-MSNs were filtered, washed, and dried in the oven at 100 °C for 20 h.

As a control, solid structure mesoporous silica (S-MSN) was synthesized using cetyltrimethylammonium bromide (CTAB) as a structure-directing agent [36]. First, 0.32 g CTAB was dissolved in 70 mL deionized water which was stirred with 30 mL ethanol and 0.5 mL ammonia at 30 °C for 1 h; subsequently, 0.5 g TEOS was added into the solution under stirring for 3 h. After the reaction, the white products were separated by centrifugation and washed once with deionized water and then twice with ethanol. The products were then dispersed in 60 mL of ethanol; 0.12 mL concentrated hydrochloric acid was added at 60 °C and was stirred for 3 h with a speed of 20 rpm. The obtained products were washed three times with deionized water and freeze-dried.

### Preparation of Amino-Chemical C-MSN-NH_2_

First, 7 mL anhydrous ethanol was added into 0.2 mL 3-aminopropyltriethoxysilane (APTES) named solution A and dispersed for 1 h. Next, 0.05 g MSN was added to 8 mL anhydrous ethanol to generate solution B. Solutions A and B were then mixed at 40 °C under constant temperature and water bath conditions for 8 h. After the reaction, the products were washed three times with anhydrous ethanol, then frozen and dried to obtain the amino-chemical C-MSN-NH_2_ powder.

### Preparation of C-MSN-FITC (MSN-F) and C-MSN-FITC-CAQK (MSN-FC)

A total of 5 μL FITC or FITC-CAQK (Zhejiang Ontores Biotechnologies Co.), 0.0020 g 1-(3-dimethylaminopropyl)-3-ethylcarbodiimide hydrochloride (EDC), and 0.0012 g *N*-hydroxysuccinimide (NHS) were added to 20 mL deionized water. The solution was dispersed for 30 min, and the pH value was adjusted to 5.5 with 0.1 M HCl, yielding solution C. Then, 10 mg C-MSN-NH_2_ was dispersed in 5 mL 0.1 M NaOH, named solution D, and the solution C was dropped slowly into solution D. Next, 0.1 M NaOH was used to adjust the pH of the solution D to 6. After reacting at ambient temperature for 6 h, the product was obtained by centrifugation and washing three times with deionized water. MSN-F or MSN-FC was obtained after freeze-drying.

### Preparation of C-MSN/FITC-CAQK/ARC-G (MSN-FC@ARC-G)

A total of 10 mg MSN-FC was added to 0.1 mg mL^−1^ ARC-G solution and soaked at 4 °C for 24 h. The sample was obtained after centrifugal freeze-drying.

### Characterization of MSNs

The morphology of MSNs after coating with gold was examined using a field emission scanning electron microscope (FESEM; Merlin, Zeiss, Germany) at an accelerating voltage of 10 kV. The particles were observed by transmission electron microscopy (HRTEM, JEM-2100F, JEOL, Japan) and analyzed by X-ray diffractometry (XRD) employing CuKα X-ray at 40 kV and 100 mA using a powder X-ray diffractometer (XRD, X’Pert PRO, PANalytical, Netherlands) and a silicon zero background plate. The Fourier transform infrared (FTIR) spectrum of MSNs was recorded using an FTIR-350 spectrometer (FTIR; AVATAR 360, Nicolet Co, USA) by the KBr pellet method. The nitrogen gas (N_2_) adsorption–desorption isotherm of MSNs was measured at − 196 °C using a specific surface area/pore size distribution analyzer under continuous adsorption conditions. The surface chemical compositions of the dried samples were analyzed by X-ray photoelectron spectroscopy (XPS; Axis Ultra DLD, Kratos, Britain).

### ARC-G Release Behavior In Vitro and In Vivo

#### Preparation of the ARC-G Mother Solution (1 mg mL^−1^)

ARC-G (100 mg) was fully dissolved in polyethylene glycol 400 (PEG 400; 26 mL) by stirring. Then, ultrapure water was added to the ARC-G-PEG solution, the volume of which was kept at 100 mL. The pH of the prepared solution was adjusted to 4.0–4.5 by adding sodium citrate and sterilized at 115 °C for 30 min.

The ARC-G-PEG 400 (26 mL) was dissolved in ultrapure water to prepare the PEG solution (100 mL); the pH was adjusted to 4.0–4.5 by adding sodium citrate and the solution was sterilized at 115 °C for 30 min.

#### In Vitro Release of ARC-G

The intravenous injection of ARC-G is highly toxic [37]; therefore, we adopted low-dose drug loading. A total of 10 mg C-MSNs was added to 10 ml ARC-G solution (0.1 mg mL^−1^) which was soaked at 4 °C for 24 h. The sample was obtained after centrifugal freeze-drying and suspended in 10 mL of phosphate-buffered saline (PBS; KeyGEN biotech, China) with mild shaking (100 rpm) in an air-bath shaker at 37 °C. At predetermined time intervals (1, 2, 4, and 6 h), release tube was centrifuged at 12,000 rpm to precipitate the microspheres, and 0.5 mL of the supernatant was extracted for a concentration test. At the same time, 0.5 mL of fresh PBS was added to the tubes. The concentration of ARC-G in the release medium was quantified by an ultraviolet spectrophotometer at 280 nm and the following calculated:

$$ {\text{Entrapment}}\;{\text{efficiency}}\; (\%) =\, \left( {{\text{total}}\;{\text{amount}}\;{\text{of}}\;{\text{ARC-G}} - {\text{free}}\;{\text{ARC-G}}} \right)/{\text{total}}\;{\text{amount}}\;{\text{of}}\;{\text{ARC-G}} \times 100\% $$

$$ {\text{Loading}}\;{\text{efficiency}}\; (\%) =\, {\text{weight}}\;{\text{of}}\;{\text{loaded}}\;{\text{ARC-G}}/{\text{total}}\;{\text{weight}}\;{\text{of}}\;{\text{nanoparticles}}\;{\text{and}}\;{\text{loaded}}\;{\text{ARC-G}} \times 100\% $$

#### In Vivo Release of ARC-G

C57BL/6J mice without spinal cord injury were fasted for 12 h but had free access to drinking water. They were then injected with 0.1 mL C-MSN-F (5 mg mL^−1^), ARC-G (0.1 mg mL^−1^), and MSN-FC@ARC-G (5 mg mL^−1^) solution via the tail vein. Before and 0.16, 1, 2, 4, and 6 h after administering the compounds, blood was taken from the venous plexus and 0.5 mL of blood was placed in a tube containing heparin sodium, vortexed, centrifuged at 6000 rpm for 10 min, and plasma was stored at − 20 °C. The release behavior of ARC-G in mice was tested by high-performance liquid chromatography (HPLC) (1260 infinity, Agilent Technologies, USA) with the following parameters: chromatographic column: Agilent HC-C18(2) column (250 × 4.6 mm, 5 μm); mobile phase: acetonitrile containing 0.1 vol% trifluoroacetic acid; flow rate: 1 mL min^−1^ injection volume: 10 μL; total running time: 30 min; test wavelength: 280 nm.

### Cell Culture

All cell-culture-related reagents were purchased from HyClone. RAW 264.7 cells (a mouse macrophage cell line, provided by the Gaolaboratory at Jinan University, China) were cultured in RPMI-1640 medium supplemented with 10% fetal bovine serum (FBS) and 1% penicillin/streptomycin in a 5% CO_2_-humidified chamber at 37 °C. Bone marrow-derived stromal cells (BMSCs) were cultured in Dulbecco’s modified Eagle’s medium with low glucose (DMEM-LG) containing 10% (v/v) FBS. Cell density was determined using a hemocytometer prior to each experiment. Cells were seeded in 96-well plates (1 × 10^4^ cells per well) with 200 μL of H-DMEM supplemented with 10% FBS and maintained in an incubator at 37 °C in a humidified atmosphere consisting of 5% CO_2_. After culturing for 12 h, the cell culture medium was replaced with 200 μL of the H-DMEM medium containing different concentrations of C-MSNs (0, 0.01, 0.03,0.06, 0.09, 0.12, and 0.15 mg mL^−1^). Five replicates were set for every sample. Cell viability was quantitatively determined using the CCK-8 assay. The absorbance at 450 nm was determined using a microplate spectrophotometer as an indicator of cell viability. The cell viability of treated cells was normalized to that of the control group (did not receive any treatment), and the following formula was used to calculate cell growth inhibition: cell viability (%) = (mean of Abs. value of treatment group/mean Abs. value of control) × 100%.

### Quantitative Real-Time RT-PCR

Total RNA was isolated using TRIzol reagent according to the manufacturer’s instructions (Invitrogen, Carlsbad, USA) and was converted into cDNA with random hexamers and PrimeScript RT Enzyme Mix (TaKaRa, Guangzhou, China). HPRT was used as an internal control. The messenger RNA (mRNA) levels of IL-17 and its related factors were analyzed through quantitative real-time RT-PCR (qRT-PCR). These IL-17-related factors were IL-17A, IL-17F, ROR-γt, IL-23R, CCR6, and granulocyte–macrophage colony-stimulating factor (GM-CSF). The mRNA levels of target genes were quantified using SYBR green mix (Bimake, Guangzhou, China) and the CFX Connect™ instrument (Bio-RAD, Guangzhou, China). The relative mRNA levels of multiple cytokine genes in each sample were displayed as 2^−ΔΔCt^ values and were representative of at least three independent experiments.

### Animals and Groups

C57BL/6J mice were purchased from the Guangdong Experimental Animal Center. All mice were female, 7–8 weeks old, and weighed 17–22 g at the time of surgery. C57BL/6J mice were fasted but could drink freely for 12 h. Then, 0.1 mL PBS, C-MSN (5 mg mL^−1^), ARC-G (0.1 mg mL^−1^), or MSN-FC@ARC-G (5 mg mL) solution was injected into the tail vein. The Jinan University Animal Ethics Committee approved this study. Animals were randomly divided into four groups. Each group was housed in a separate cage and had free access to water and a normal diet. The animals were kept in a room with an ambient temperature of 20–25 °C and relative humidity of 60%.

### Contusive SCI Model

Contusive SCI was performed using a New York University Impactor as described [38]. In brief, mice were anesthetized with pentobarbital (50 mg kg^−1^ intraperitoneally) and underwent a laminectomy at the T11–12 level. The exposed dorsal surface of the cord was subjected to a weight drop injury using a 10-g rod dropped from a height of 6.25 mm.

### Mice Fluorescence Imaging

After the mice were injected with MSN–FC@ARC-G and MSN–F for 24 h, the animals were placed in Bruker In Vivo Imager, anesthetized with isoflurane, and images were collected with 470 nm excitation wavelength, 535 nm emission wavelength, and three seconds exposure time. The background noise of images was removed by the analysis software. Immediately, the mice were euthanized, and tissues (including heart, liver, spleen, lung, kidney, and spine) were obtained after surgery. The distribution of the nanoparticle in the tissues was analyzed under the same imaging parameters.

### Behavioral Assessment

Recovery after SCI was evaluated using the Basso Mouse Scale (BMS) assessment scores [39]. Two blinded expert observers evaluated the mice on the day of surgery, and at 1, 3, and 5 days, 1, 2, 3, 4, 5, and 6 weeks after surgery. Scores from the two observers were averaged. Also, recovery was assessed according to published methods using the CatWalkXT 9.1 automatic quantitative gait analysis system (Noldus Company, Wageningen, Netherlands). Briefly, mice were trained five times a day for 1 week before surgery to walk in a single direction the entire length of a darkened CatWalk chamber placed in a quiet room. Six weeks after surgery, mice were assessed five times using the same system. The gait regularity index and hind max contact area were recorded. The regularity index calculated as the number of normal step sequence patterns multiplied by four and divided by the total amount of paw placements; that value is ~ 100% in normal animals.

### Motor-Evoked Potentials

To evaluate SCI recovery, the motor-evoked potentials (MEP) were assayed at 6 weeks after treatment following previously described methods [40]. First, the mice were anesthetized using a compound anesthetic (3.0 mL kg^−1^). Then, a stimulation electrode was applied to the rostral ends of the surgical spinal cord. The recording electrode was placed in the gastrocnemius, and the reference electrode was placed in the paravertebral muscles between the stimulation point and recording point. The ground electrode was placed on the tail. A single square wave stimulus of 0.5 mA, 0.5 ms in duration, 2 ms time delay, and 1 Hz was used. The amplitude was measured from the initiation point of the first response wave to its highest point. All potentials were amplified and obtained using a digital oscilloscope (Chengdu Instrument Factory, Chengdu, China).

### Nissl Staining and Immunofluorescence

At 6 weeks after surgery, 8 mice were selected from each group, anesthetized, and fixed by perfusion with 4% paraformaldehyde in 0.1 M PBS (pH 7.4). The T11 vertebra was exposed as described above, and 1 cm spinal cord specimens around the transected site were collected. The injured sites, the anterior and posterior ends, and the dorsal and ventral sides were marked. Specimens were fixed for 24 h with 4% neutral paraformaldehyde, embedded, and sectioned using a cryostat with a sagittal plane thickness of 20 μm. To measure Nissl body density and shape, frozen sections were stained with Nissl reagent (Goodbio, Wuhan, China) strictly according to the manufacturer’s instructions. Frozen sections were also probed with rabbit antibodies against the glial fibrillary acidic protein (GFAP, diluted 1:1000; Abcam, Cambridge, UK) and then labeled with Alexa Fluor 488-conjugated goat anti-rabbit IgG (diluted 1:500; Abcam). Images were obtained using an inverted fluorescence microscope (Leica DM1000, German). Fluorescence intensity was calculated using Image J (National Institutes of Health, Bethesda, MD, USA). For quantitative analysis of cavity volume, serial spinal cord sections stained with GFAP were three-dimensionally reconstructed. A total of five serial transverse spinal cord sections equally spaced 200 µm apart were used to create a three-dimensional image corresponding to a 1 cm-long spinal cord segment. The lesion cavity and the spinal cord were three-dimensionally reconstructed using a microscope attached to a Neurolucida system (MBF Bioscience). The Neurolucida software calculated the volumes of the cystic cavities automatically [3].

### Inflammatory Cytokine Analysis

For analyzing the IL-17 in spleens, 3 days post-injury mice were perfused with phosphate-buffered saline (PBS). Spleens were removed by insufflation and dissociated by gently grinding the tissue into a single-cell suspension. Infiltrated cells were enriched by Percoll gradient centrifugation and resuspended in staining buffer.

At 1 week after treatment, a 10-mm segment of cord centered at T11 was removed and quickly frozen in an ethanol-dry-ice bath. Samples were stored at − 80 °C. The tissue samples were later homogenized in 200 μL of ice-cold cell lysis buffer (Beyotime Inc., China) containing enzyme inhibitors and centrifuged at 10,000×*g* for 10 min at 4 °C; then, the supernatants were frozen at − 80 °C. Protein levels were determined using the BCA protein assay as described by the manufacturer (Beyotime).

The levels of IL-17, IL-1α, IL-6, granulocyte colony-stimulating factor (G-CSF), MCP-1, and MIP-1β in mouse spinal cords were analyzed with the Bio-Plex system (Bio-Rad, Hercules, CA, USA) using a 23-Plex Cytokine Array Kit (Catalog No. M60009RDPD).

## Results

### Construction of the Nanosystem

**Fig. 1:**
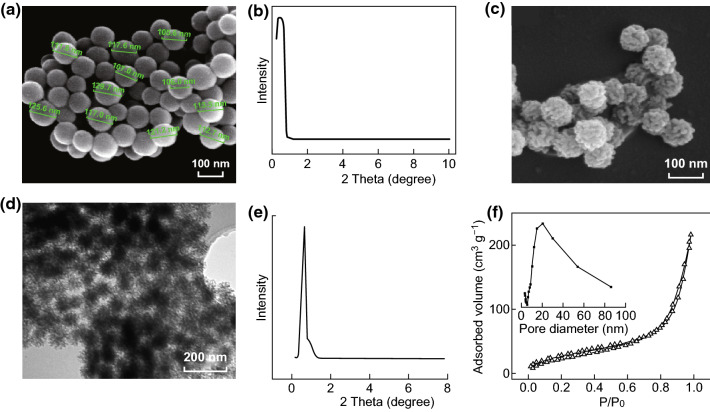
Physicochemical characterization of the MSN. **a** SEM image and **b** XRD pattern of S-MSN. **c** SEM image, **d** TEM image, **e** XRD pattern, **f** N_2_ adsorption–desorption isotherm of C-MSN

The SEM and TEM images (Fig. 1c, d) showed that the particle size of C-MSN was around 60–120 nm. Thin bridges featuring porous channels between mesoporous particles formed inside the C-MSN particles which were apparently held together in aggregates. As a comparison, S-MSN particles were prepared (Fig. 1a), which showed dense structures. Figure 1e, b displays SAXRD patterns of C-MSN and S-MSN with distinct diffraction peaks at 0–1, indicating that the MSN had a partially ordered mesostructure. The peak in Fig. 1e appears sharper than that in Fig. 1b. The SAXRD results were consistent with the morphology results (Fig. 1a, c, d). As shown in Fig. 1f, the C-MSN had a classical type IV N_2_ adsorption–desorption isotherm with well-defined steps at relative pressures (*P*/*P*_0_) of 0.2–0.4 and 0.8–1.0 for C-MSNs. This suggested that C-MSN had uniform mesoporous channels and relatively narrow pore size distribution, which was also supported by Fig. 1c, d consistent with the results obtained from SEM and TEM imaging. Surprisingly, C-MSN had two most likely pore diameters of approximately 3.0 and 19.5 nm (Fig. 1f) as suggested by two steps of its desorption isotherm implying the presence of multiple probable mesoporous structures. Also, C-MSN had specific surface areas and cumulative pore volumes (82.880 m^2^ g^−1^ and 0.3210 cm^3^ g^−1^, respectively).

**Fig. 2:**
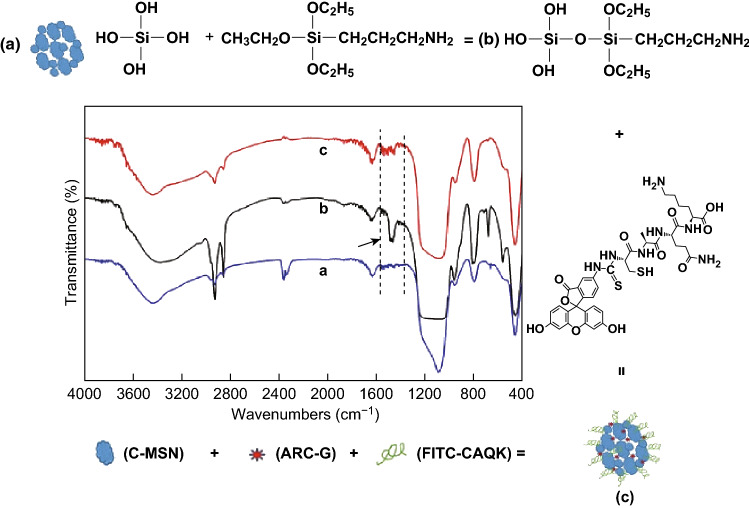
The FTIR spectra of nanomaterials. **a**–**c** Different grafting steps: **a** raw C-MSN, **b** C-MSN-NH_2_, and **c** C-MSN-FITC-CAQK

Figure 2 presents the FTIR spectra of C-MSNs at different grafting steps. Raw C-MSNs (Fig. 2a) showed the typical absorption peaks approximately 1095, 794–470, and 1640 cm^−1^, which were assigned to Si–O–Si, Si–O stretching peak, and H–OH bending vibration peak, respectively. Figure 2b shows the typical bending vibration peak of −NH_2_ approximately 1500 cm^−1^ after grafting with -NH_2_. As displayed in Fig. 2c, the FITC-CAQK was grafted to C-MSNs with -NH_2_ following which the typical bending vibration peak of -NH_2_ approximately 1500 cm^−1^ disappeared.

**Fig. 3:**
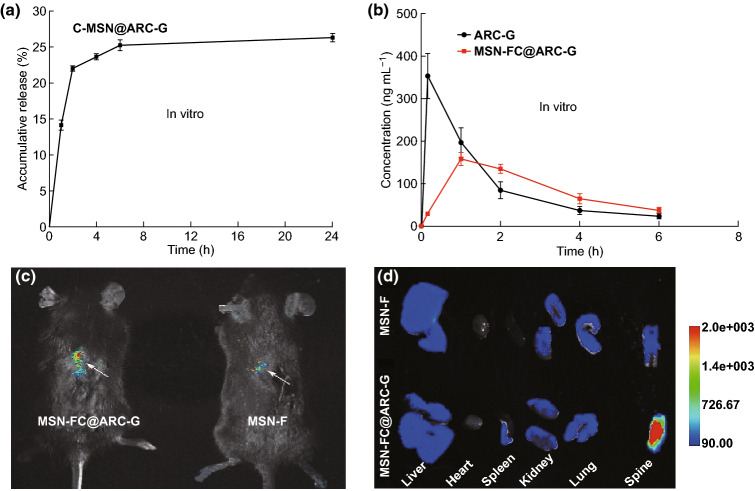
Drug release behavior of nanomaterials. **a** In vitro and **b** in vivo. Concentration of ARC-G was determined by HPLC (*n* = 4/group). **c** Targeting ability of MSN-FC@ARC-G to the injured spinal cord. Representative NIRF images of mice 1 day after injection. Arrows point to the region of the injured spinal cord. **d** Representative NIRF images of the excised organs from mice

Drug entrapment and loading efficiency of the nanosystem were calculated as 31.21 + 3.6% and 1.56 + 0.25%, respectively. Figure 3a shows the ARC-G releasing behavior of C-MSN@ARC-G in PBS. ARC-G released from C-MSN slowly, and the cumulative release ratio was only 22.02% at 2 h. After 6 h, the cumulative release ratio was up to 25.27%, indicating that the nanosystem demonstrated sustained drug release behavior in vitro. The more slowly releasing behavior was obtained. Figure 3b displays the ARC-G releasing behavior of MSN-FC@ARC-G in vivo. The initial drug concentration of the ARC-G group was greater than that of the MSN-FC@ARC-G group at 1 h, but the MSN-FC@ARC-G group showed sustained drug release behavior after intravenous injection. To examine the ability of MSN-FC@ARC-G to target the injured spinal cord tissue in vivo, MSN-F and MSN-FC@ARC-G were intravenously injected into mice tail veins 1 day after the injury. After 24 h, the spinal cord was dissected and analyzed by NIRF imaging (Fig. 3c, d). The fluorescence intensity of the lesion was significantly higher after MSN-FC@ARC-G administration compared with MSN-F (Fig. 3c). These results indicated that CAQK significantly enhanced the tropism to the region of the injured spinal cord.

### In Vitro Cytotoxicity Assay

**Fig. 4:**
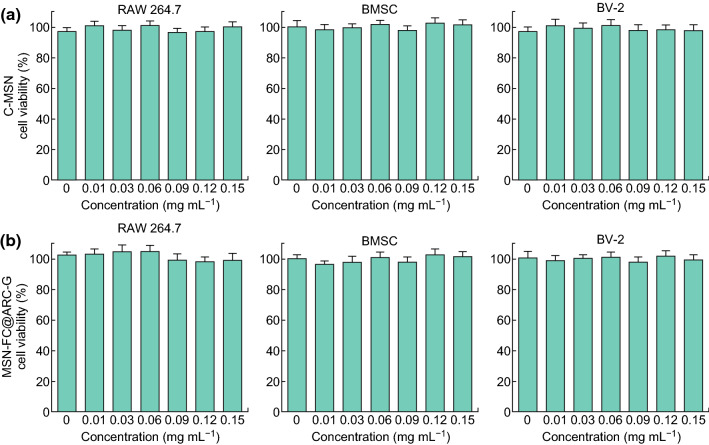
In vitro cytotoxicity assay. **a–d** Cell viability of RAW 264.7, BMSC, and BV-2 after incubation with various concentrations of C-MSN and MSN-FC@ARC-G

Before exploring the in vivo performance, biocompatibility of C-MSN and MSN-FC@ARC-G was evaluated by the CCK-8 assay (Fig. 4). Our results demonstrated that C-MSN and MSN-FC@ARC-G did not show any obvious toxic effects to RAW 264.7 cells and BMSCs after incubation for 48 h even at a high concentration of up to 0.15 mg mL^−1^ which surpassed that used in the following experiments. Figure 4 shows no detectable acute toxic effects of C-MSN and MSN-FC@ARC-G.

### Side Effects and Systemic Toxicity

**Fig. 5:**
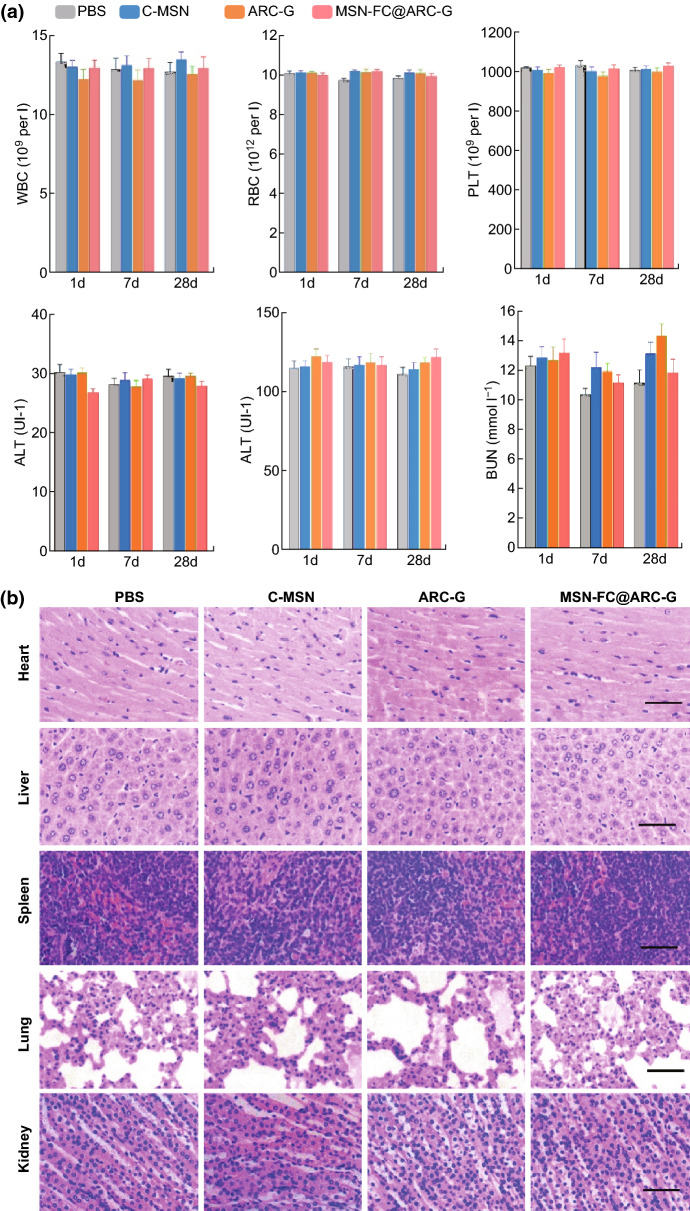
Absence of side effects and systemic toxicity following intravenous injection of C-MSN, ARC-G, and MSN-FC@ARC-G. **a** Acute (day 1 and day 7) and long-term (day 28) toxicity of C-MSN upon systemic administration; **b** Histological analysis of heart, liver, spleen, lung, and kidney after 28 days (scale bar: 100 μm). WBC, white blood cells; RBC, red blood cells; PLT, platelets; ALT, alanine transaminase; AST, aspartate transaminase; BUN, blood urea nitrogen (scale bar: 50 µm)

The overall toxicity to mice of intravenously administered C-MSN, ARC-G, and MSN-FC@ARC-G (ARC-G concentration: 1 mg kg^−1^; 1, 7, and 28 days post-injection) compared to control animals (PBS) is shown in Fig. 5. No difference was noted among the groups concerning white blood cells, red blood cells, or platelets. The levels of alanine transaminase, aspartate transaminase, and blood urea nitrogen were not increased in the experimental group. MSN-FC@ARC-G did not induce any increase or decrease in white blood cells, red blood cells, or platelets. Similarly, no differences in alanine transaminase, aspartate transaminase, or blood urea nitrogen were observed between the experimental group animals and the controls (PBS). These results indicated that MSN-FC@ARC-G did not induce obvious inflammation and affect the blood chemistry of mice (Fig. 5a). Finally, the corresponding histological changes of organs are shown in Fig. 5b; the heart, liver, spleen, lung, and kidney had no morphological damage 28 days post-injection of MSN-FC@ARC-G (Fig. 5b) indicating good biocompatibility of these nanocarriers.

### MSN-FC@ARC-G Promoted Locomotor Functional Recovery after SCI

**Fig. 6:**
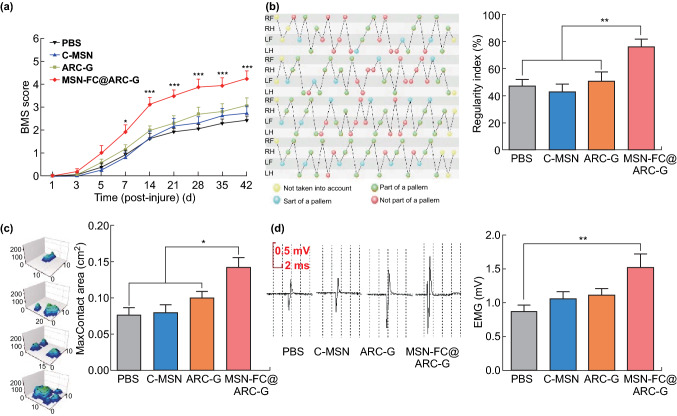
a BMS scores at different time-points after spinal cord contusion (*n* = 8 mice per group; repeated measures of ANOVA with Bonferroni’s post hoc correction). **b** Regularity index. **c** hind max contact area was analyzed using the CatWalk XT automated quantitative gait analysis system (*n* = 8 mice per group). **d** Examples and the statistic histogram of motor-evoked potential (MEP) recordings from mice 8 weeks post-surgery (*n* = 8 mice per group) (**b**, **c**, and **d**) One-way ANOVA with Tukey’s multiple comparisons test; Mean ± SEM; *P < 0.05; **P < 0.01; ***P < 0.001

To evaluate the therapeutic effect of the nanoparticles, mice were treated with PBS, C-MSN, ARC-G, or MSN-FC@ARC-G. After contusion, all four groups of animals sustained complete paraplegia with no observable hindlimb movement by the BMS assessment. The MSN-FC@ARC-G group recovered gradually, with the BMS index increasing from 1 week post-injury and peaking at 6 weeks post-injury (with an average of 4.25 ± 0.31, *n* = 8) (Fig. 6a). In contrast, functional recovery in other groups was significantly slower with a limited increase in the BMS index of approximately 2.5 at 6 weeks post-injury (Fig. 6a). This significant difference was also apparent in an increased regularity index (calculated as the number of normal step sequence patterns multiplied by four and divided by the total amount of paw placements; ~ 100% in normal animals) and enlarged hind max contact area in the MSN-FC@ARC-G group compared with control animals 6 weeks after injury (MSN-FC@ARC-G, 76.44 ± 5.51 vs. PBS, 47.73 ± 4.29 vs. MSN 43.19 ± 5.36 vs. ARC-G, 51.12 ± 6.38, n = 8; Fig. 6b and MSN-FC@ARC-G, 0.14 ± 0.01 vs. PBS, 0.08 ± 0.01 vs. MSN, 0.08 ± 0.01 vs. ARC-G, 0.10 ± 0.01, *n* = 8; Fig. 6c). To confirm these results, electromyography of the bicep flexor cruris was recorded at 6 weeks post-injury. The results showed that the amplitudes of the motor-evoked potential (MEP) were significantly higher in the MSN-FC@ARC-G group than in other groups (MSN-FC@ARC-G, 1.53 ± 0.20 vs. PBS, 0.87 ± 0.09 vs. MSN, 1.06 ± 0.10 vs. ARC-G, 1.11 ± 0.09, *n* = 8; Fig. 6d).

### MSN-FC@ARC-G Reduced Lesion Cavities and Protected Motor Neurons

**Fig. 7:**
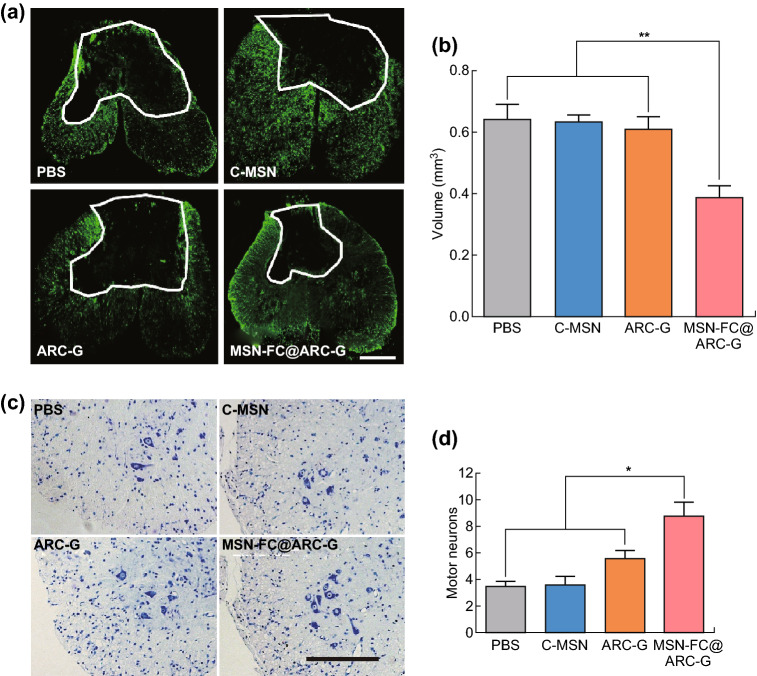
**a**, **b** Representative injury sites labeled with anti-GFAP antibodies and the statistic histogram of lesion volumes in the four groups (*n* = 8 mice per group; scale bar: 250 µm). **c**, **d** Survival of motor neurons immunostained with Nissl staining in the spinal cord ventral horn (VH) 8 weeks after SCI. (*n* = 8 mice per group; Scale bar: 250 µm) and one-way ANOVA with Tukey’s multiple comparisons tests; Mean ± SEM; *P < 0.05; **P < 0.01

Improved functional recovery suggested that there was a smaller damage volume in the MSN-FC@ARC-G-treated mice than in the control animals. We measured the cavity of the lesion center using anti-GFAP immunostaining at 6 weeks post-injury. The cavities were significantly smaller in the MSN-FC@ARC-G group than in other groups (MSN-FC@ARC-G, 0.39 ± 0.04 vs. PBS, 0.64 ± 0.05 vs. C-MSN, 0.63 ± 0.02 vs. ARC-G, 0.61 ± 0.04, *P* < 0.01, *n* = 6; Fig. 7a, b). The number of surviving motor neurons, which are responsible for functional recovery following SCI, was also counted using Nissl staining. There were more surviving motor neurons in mice that received MSN-FC@ARC-G treatment than in other groups (MSN-FC@ARC-G, 8.88 ± 1.03 vs. PBS, 3.50 ± 0.33 vs. C-MSN, 3.63 ± 0.57 vs. ARC-G, 5.63 ± 0.53, *P* < 0.05, *n* = 6; Fig. 7c, d). These results indicated that MSN-FC@ARC-G improved functional recovery after SCI.

### MSN-FC@ARC-G Reduced IL-17 Expression In Vivo

**Fig. 8:**
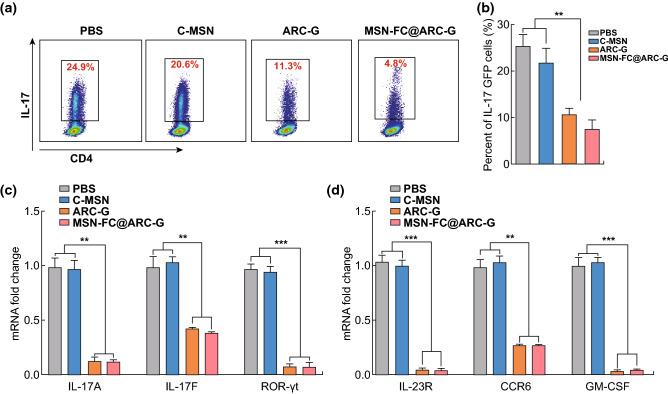
**a**, **b** Flow cytometry analysis of the expression of IL-17 in CD4 + T cells from the spleen (*n* = 8 mice per group). One-way ANOVA with Tukey’s multiple comparisons tests; Mean ± SEM; **P < 0.01. **c**, **d** qRT-PCR analysis of the expression of IL-17-related mRNA in the spinal cord (*n* = 8 mice per group). One-way ANOVA with Tukey’s multiple comparisons tests; Mean ± SEM; **P < 0.01; ***P < 0.001

Flow cytometry analysis was performed to determine whether the neuroprotection effect of MSN-FC@ARC-G on SCI was associated with changes in IL-17, which is known to induce myelin destruction, neuronal death, and astrocyte toxicity in different inflammatory conditions of the central nervous system. After SCI, the mice were injected with PBS, C-MSN, ARC-G, or MSN-FC@ARC-G, and the spleens were extracted 1 day later. The results clearly showed that MSN-FC@ARC-G significantly reduced the expression of IL-17 (Fig. 8). Next, qRT-PCR was used to further analyze the expression of IL-17-related mRNA. As shown in Fig. 8c, d, IL-17A, IL-17F, ROR-γt, IL-23R, CCR6, and GM-CSF were inhibited in the spleens of MSN-FC@ARC-G-treated mice. Thus, the expression of IL-17 signature cytokines in peripheral lymphoid organs (spleen) of mice with SCI was inhibited by MSN-FC@ARC-G compared with the other groups.

### MSN-FC@ARC-G Reduced Expression of IL-17-Related Inflammatory Cytokines

**Fig. 9:**
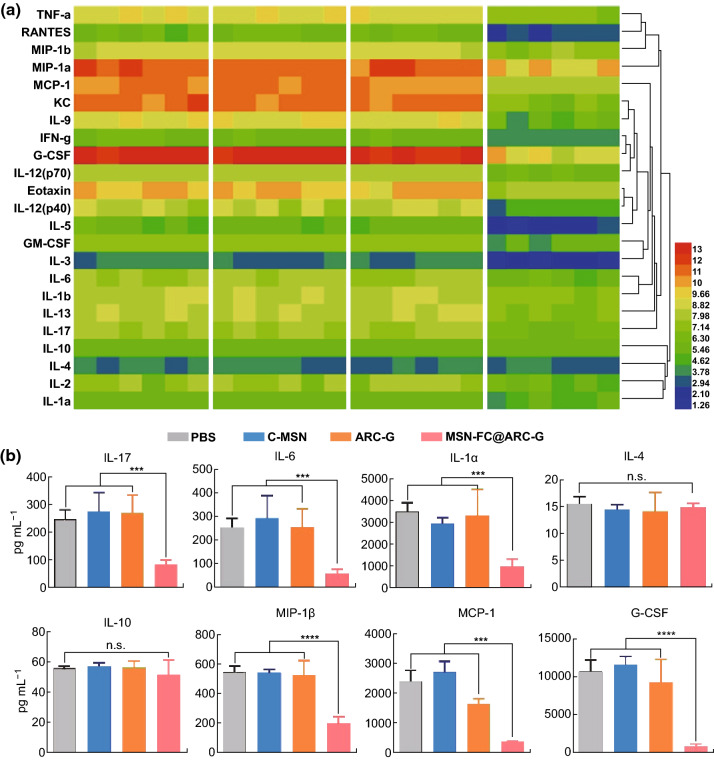
**a** Pro-inflammatory cytokines in spinal samples were analyzed using the Bio-Plex system (Bio-Rad, Hercules, CA) 24 h after SCI. Different colors indicate the protein levels from low (blue) to high (red) representing the fold change. **b** Protein levels of IL-17, IL-1α, IL-6, G-CSF, MCP-1, and MIP-1β were significantly decreased in the MSN-FC@ARC-G-treated group compared with the other three groups (*n* = 6 mice per group). One-way ANOVA with Tukey’s multiple comparisons tests; Mean ± SEM; ***P < 0.001; ****P < 0.0001

To determine whether MSN-FC@ARC-G modulated inflammation and wound healing, pro-inflammatory cytokines were investigated, and the protein expression levels of inflammatory mediators associated with IL-17 were assessed. The MSN-FC@ARC-G-treated mice showed significantly reduced pro-inflammatory cytokine levels of IL-17, IL-1α, IL-6, G-CSF, MCP-1, and MIP-1β (Fig. 9). Multiple IL-17 receptors (IL-17 ra, IL-17 rb, IL-17 rc, and IL-17 re) are expressed on astrocyte membranes; therefore, IL-17 has been shown to exert a strong influence on astrocytes in the central nervous system, especially in ischemia, inflammation, and degenerative diseases [41]. We, therefore, verified whether the nanomaterial system plays a role in regulating astrocytes.

### MSN-FC@ARC-G Regulated the Activation of Astrocytes

**Fig. 10:**
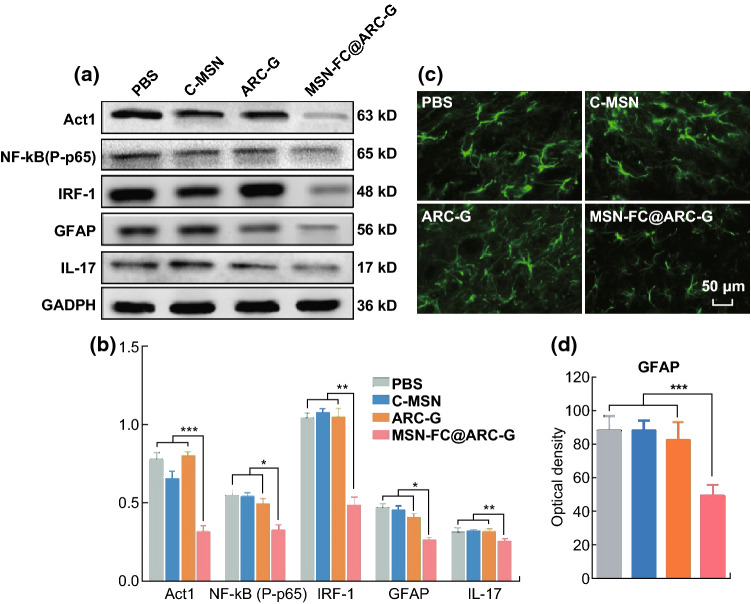
**a** Western blotting and mRNA expression of IL-17, GFAP, and related proteins in four groups of mice with indicated treatments (*n* = 5 mice per group). **c** Immunofluorescence detection of the astrocyte activation in four groups of mice with indicated treatments (*n* = 8 mice per group; distance from injury site 1 mm proximal at the anterior horn of spinal cord). (**b**, **d**) One-way ANOVA with Tukey’s multiple comparison tests; Mean ± SEM; *P < 0.05; **P < 0.01; ***P < 0.001

Western blotting indicated that MSN-FC@ARC-G significantly decreased the expression of proteins involved in the Act1/NF-κb/IRF-1 signaling pathway (Fig. 10a, b). Moreover, immunofluorescence analysis showed that MSN-FC@ARC-G could inhibit the activation of astrocytes (GFAP expression was enhanced with the increased activation of astrocytes) (Fig. 10c, d). These results indicated that the molecular mechanisms underlying the therapeutic effect of MSN-FC@ARC-G on mouse SCI involve IL-17, which regulates the Act1/NF-κb/IRF-1 signaling pathway of astrocytes.

## Discussion

SCI can lead to severe motor, sensory, and autonomic dysfunction [42]. Drug delivery remains the main challenge of SCI drug development due to the fast metabolism and/or rapid blood clearance of most SCI drugs, as well as poor diffusion through the blood–spinal cord barrier (BSCB) [43]. It has been suggested that nanotechnologies may promote spinal cord delivery and efficacy of drugs because of an improved pharmacokinetic profile and better neurovascular unit access [12, 24–27]. However, many of these nanocarriers require complex functional design to achieve targeted delivery of drugs which may restrain their pharmaceutical development [44].

In our study, we used C-MSN as the drug carrier; and the particle size of C-MSN was designed to be approximately 100 nm (Figs. 1 and S1). We developed the C-MSN/ARC-G/CAQK composite nanoplatform to enable site-specific drug delivery to the spinal cord. To achieve a functional nanocarrier design, the FITC fluorescent molecule, and a CAQK peptide, which could selectively identify the damaged central nervous system [33, 34], were grafted onto the C-MSN nanoparticles. The verification results are shown in Figs. S2 and S3 by FTIR and XPS analysis. A previous study reported that the size threshold for therapeutic nanoparticle localization to the spinal cord after contusion injury is below 200 nm [45]. The nanocarriers we synthesized meet this size requirement (Fig. 1c, d). Importantly, the in vivo live imaging showed that the nanosystem (MSN-FC@ARC-G) efficiently reached the spinal cord injury site (Fig. 3c). MSN-F did not have any targeting ligands, but the fluorescence signals still accumulated near the injured spinal cord. The possible reason is that the blood–brain barrier will be open temporarily after SCI. Even if there is no targeting effect, MSN-F of 100 nm will penetrate part of the injured spinal cord tissue through blood vessels. That is why MSN-F accumulated near injured spinal cord, as shown in Fig. 3c.

Some authors have reported that the intravenous injection of ARC-G is highly toxic [37]; low-dose drug loading was achieved in this work, and it was found that the loading efficiency of the nanosystem was only 1.56 + 0.25%. Due to the porous channels formed between mesoporous particles inside the C-MSN particles (Fig. 1c, d), drug release of C-MSN was fast during the first 2 h, and the cumulative release ratio by the end of 2 h was only 22.02%. After 6 h, the cumulative release ratio increased to 25.27%, indicating that the nanosystem demonstrated sustained drug release behavior in vitro. The in vivo releasing behavior of MSN-FC@ARC-G also showed sustained drug release after intravenous injection (Fig. 3a, b).

BSCB disruption is an important contributor to secondary injury following SCI, and therapies to restore BSCB functionality are under investigation for neuroprotection [46]. The localized permeability of the BSCB and the delayed onset of secondary injury provide a window of opportunity for therapeutic intervention. Our results suggested that the duration of BSCB impairment was at least 24 h (Fig. 3c, d). Within this time window, CAQK targeting can be an effective drug delivery approach with two advantages. First, the peptide can access and bind to its target allowing accumulation of the payload at the site of injury. The second important factor is the retention effect. By binding to its target, CAQK can retain the drug in the injured microenvironment by minimizing its washout [34, 47]. Thus, the targeting approach in this study encompasses the critical period of healing, which may provide a more lasting therapeutic effect.

Most CNS medicines have significant limitations due to their side effects [48]. The results of the biocompatibility experiment we conducted are shown in Figs. 4 and 5. First, the in vitro cell experiments showed no toxic effects of the nanomaterials. Subsequently, we investigated the overall toxicity of the intravenously administered nanocarrier in mice compared to control animals injected with PBS. No differences were noted between the two groups concerning white blood cell count, red blood cell count, and other hematological parameters (Fig. 5a); the heart, liver, spleen, lung, and kidneys also showed no morphological damage after nanocarrier administration at 28 days post-injection (Fig. 5b). These results clearly showed that the nanocarrier can be considered safe upon systemic intravenous administration at the therapeutic dose of 5 mg mL^−1^.

We investigated the therapeutic effect of the nanoplatform on SCI. BMS results showed that the mice treated with MSN-FC@ARC-G recovered better than those in other groups (Fig. 6a). Additionally, the MSN-FC@ARC-G group was better than the control group in both the hind max contact area and the gait index (Fig. 6b, c). The electrophysiological test results also showed that the nerve conduction function of the MSN-FC@ARC-G group was better than that of the control group (Fig. 6d). To more objectively evaluate the therapeutic effect of MSN-FC@ARC-G, morphological analysis of the SCI site was performed (Fig. 7). The cavity of the lesion center was measured using anti-GFAP immunostaining at 6 weeks post-injury. The cavities were significantly smaller in the MSN-FC@ARC-G-treated mice than in other groups (Fig. 7a, b). Next, the number of surviving motor neurons, which are responsible for functional recovery following SCI, was assessed. The results showed more surviving motor neurons in mice with MSN-FC@ARC-G treatment (Fig. 7c, d). These results indicated that MSN-FC@ARC-G effectively improved the nerve function of spinal cord injury; however, its underlying mechanism is not clear.

It has been reported that the levels of inflammatory factors were increased dramatically within 24 h after SCI injury [49] and played a key role in the secondary injury after SCI, leading to spinal cord demyelination and neuronal death [50]. The anti-inflammatory and neuroprotective role of ARC-G has previously been reported in a mechanical trauma injury model [51] that could reduce the expression of IL-17, IL-1, and TNF-α [52]. The decreased expression of IL-17 could effectively promote the repair of central nerve injury [53, 54].

To explore the molecular mechanism of ARC-G in the treatment of spinal cord injury, we conducted a series of biological experiments. First, the expression of IL-17 after SCI in the CD4 + T cells of the spleen was analyzed through flow cytometry and q-PCR. The results showed that there was a significant reduction in IL-17 and related factors in the ARC-G and MSN-FC@ARC-G-treated groups (Fig. 8a–d), indicating that IL-17 plays an important role in and participates in multiple immune responses. To verify whether the down-regulation of IL-17 was accompanied by the attenuation of the associated inflammatory factors, multi-factor high-throughput detection of the local spinal cord was carried out. At 24 h after spinal cord injury, 5-mm segments centered on the injury site were collected for luminex analysis [55]. The results showed that the expression of IL-17 and IL-17-related inflammatory factors was downregulated (Fig. 9). These results indicated that both ARC-G and MSN-FC@ARC-G could reduce the expression of IL-17 and related factors in the spleen (Fig. 8). MSN-FC@ARC-G could reduce IL-17-related inflammatory factors in local SCI due to its targeting ability, whereas ARC-G could not (Fig. 9). Taken together, these data suggested that MSN-FC@ARC-G effectively improved local inflammation of the spinal cord injury microenvironment.

**Scheme 2:**
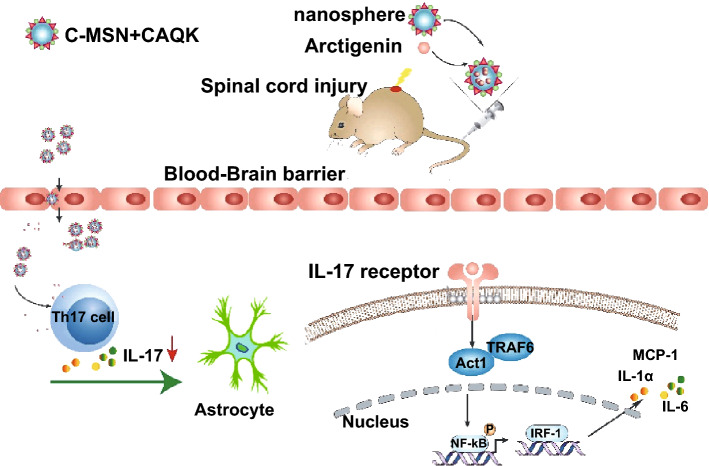
Immune responses and neuroinflammation are critically involved in SCI

Astrocytes, as an essential component of the central nervous system, play an important role in supporting the neurons [56]. Under stress conditions, such as spinal cord injuries, traumatic brain injury, stroke, infection, or degenerative diseases [57], astrocytes can be rapidly activated accompanied by changes in their morphology and function. These changes include excessive expression of chondroitin sulfate promoting the formation of glial scars [58] and the ability to adjust the balance of extracellular ions, repair the blood–brain barrier, and secrete neurotrophic factors, such as NGF, NT3, and GDNF, which can quickly remove cytotoxic factors and promote the functional recovery of spinal cord neurons [59]. A previous study regulated the function of astrocytes to prevent the excessive expression of GFAP for the treatment of spinal cord injury [60]. Multiple IL-17 receptors (IL-17 RA, IL-17 RB, IL-17 RC, and IL-17 RE) can be expressed on the astrocyte membrane, and IL-17 has a significant impact on astrocytes in the central nervous system, especially in ischemia and inflammation-degenerative diseases [41]. In vitro experiments have shown that IL-17A could stimulate human or mouse astrocytes to secrete a large number of inflammatory factors and chemokines (IL-6, TNF-α, CCL2, CCL3, CCL20, CXCL1, and CXCL2) [61]. Besides, the Act1/IRF-1 signaling pathway plays an important role in the activation of astrocytes [62]. With the upregulation of IL-17, Actl binds to the IL-17R, stimulating TAKl and IKK kinase to activate NF-κB and promote the release of inflammatory cytokines by astrocytes [63]. Our results indicated that blocking the expression of IL-17 inhibited the activation of astrocytes, and Western blotting showed that the protein expression associated with the Act1/IRF-1 signaling pathway decreased (Fig. 10). Considering the role of IL-17 in diseases associated with the central nervous system, and in light of our previous results [16], it seems plausible that the role of MSN-FC@ARC-G in the pathophysiological process of SCI was achieved by regulating the function of astrocytes (Scheme 2).

## Conclusion

In this study, we constructed a nanosystem of mesoporous silica + CAQK + ARC-G. We have shown that this system has good biocompatibility, can be released sustainably in vivo, and the “time window” of drug treatment is prolonged. More significantly, it can target SCI and penetrate the blood–spinal cord barrier due to its 100 nm particle size. Furthermore, this nano-drug platform can repair the local micro-environmental damage, specifically by reducing the expression of IL-17 and IL-17-related inflammatory factors, thus protecting the neurons and promoting the recovery of SCI.

## Electronic supplementary material

Below is the link to the electronic supplementary material.

Supplementary material 1 (PDF 292 kb)

## References

[1] Rubiano AM, Carney N, Chesnut R, Puyana JC (2015). Global neurotrauma research challenges and opportunities. Nature.

[2] Rust R, Kaiser J (2017). Insights into the dual role of inflammation after spinal cord injury. J. Neurosci..

[3] Sun G, Yang S, Cao G, Wang Q, Hao J (2018). Γδ t cells provide the early source of IFN-γ to aggravate lesions in spinal cord injury. J. Exp. Med..

[4] Sun G, Li G, Li D, Huang W, Zhang R, Zhang H, Duan Y, Wang B (2018). HucMSC derived exosomes promote functional recovery in spinal cord injury mice via attenuating inflammation. Mater. Sci. Eng., C.

[5] Li J, Deng J, Yuan J, Fu J, Li X (2017). Zonisamide-loaded triblock copolymer nanomicelles as a novel drug delivery system for the treatment of acute spinal cord injury. Int. J. Nanomed..

[6] Singh PL, Agarwal N, Barrese JC, Heary RF (2012). Current therapeutic strategies for inflammation following traumatic spinal cord injury. Neural Regen. Res..

[7] Bydon M, Lin J, Macki M, Gokaslan ZL, Bydon A (2014). The current role of steroids in acute spinal cord injury. World Neurosurg..

[8] Tang X, Zhuang J, Chen J, Yu L, Hu L, Jiang H, Shen X (2011). Arctigenin efficiently enhanced sedentary mice treadmill endurance. PLoS ONE.

[9] Hayashi K, Narutaki K, Nagaoka Y, Hayashi T, Uesato S (2010). Therapeutic effect of arctiin and arctigenin in immunocompetent and immunocompromised mice infected with influenza a virus. Biol. Pharm. Bull..

[10] Machado FB, Yamamoto RE, Zanoli K, Nocchi SR, Novello CR (2012). Evaluation of the antiproliferative activity of the leaves from arctium lappa by a bioassay-guided fractionation. Molecules.

[11] Gadani SP, Walsh JT, Smirnov I, Zheng J, Kipnis J (2015). The glia-derived alarmin IL-33 orchestrates the immune response and promotes recovery following CNS injury. Neuron.

[12] Papa S, Caron I, Erba E, Panini N, De Paola M (2016). Early modulation of pro-inflammatory microglia by minocycline loaded nanoparticles confers long lasting protection after spinal cord injury. Biomaterials.

[13] Pomeshchik Y, Kidin I, Korhonen P, Savchenko E, Jaronen M (2015). Interleukin-33 treatment reduces secondary injury and improves functional recovery after contusion spinal cord injury. Brain Behav. Immun..

[14] Li W, Zhang Z, Zhang K, Xue Z, Li Y (2016). Arctigenin suppress TH17 cells and ameliorates experimental autoimmune encephalomyelitis through AMPK and PPAR-γ/ROR-γt signaling. Mol. Neurobiol..

[15] Zong S, Zeng G, Fang Y, Peng J, Tao Y, Li K, Zhao J (2014). The role of IL-17 promotes spinal cord neuroinflammation via activation of the transcription factor STAT3 after spinal cord injury in the rat. Mediators Inflamm..

[16] Sun G, Hu X, Zhang G, Sun C, Zhang R, Tang S, Lin Y, Li Z (2018). Arctigenin suppresses inflammation and plays a neuroprotective effect in mice with spinal cord injury. Int. J. Clin. Exp. Med..

[17] Kabu S, Gao Y, Kwon BK, Labhasetwar V (2015). Drug delivery, cell-based therapies, and tissue engineering approaches for spinal cord injury. J. Control. Release.

[18] Limongi T, Tirinato L, Pagliari F, Giugni A, Allione M, Perozziello G, Candeloro P, Di Fabrizio E (2016). Fabrication and applications of micro/nanostructured devices for tissue engineering. Nano-Micro Lett..

[19] Liu M, Zeng X, Ma C, Yi H, Ali Z (2017). Injectable hydrogels for cartilage and bone tissue engineering. Bone Res..

[20] Cheng C, Li S, Thomas A, Kotov NA, Haag R (2017). Functional graphene nanomaterials based architectures: biointeractions, fabrications, and emerging biological applications. Chem. Rev..

[21] Yang Y, Ma L, Cheng C, Deng Y, Huang J (2018). Nonchemotherapic and robust dual-responsive nanoagents with on-demand bacterial trapping, ablation, and release for efficient wound disinfection. Adv. Funct. Mater..

[22] Mathiyazhakan M, Wiraja C, Xu C (2017). A concise review of gold nanoparticles-based photo-responsive liposomes for controlled drug delivery. Nano-Micro Lett..

[23] Li G, Chen Y, Zhang L, Zhang M, Li S, Li L, Wang T, Wang C (2017). Facile approach to synthesize gold nanorod@polyacrylic acid/calcium phosphate yolk–shell nanoparticles for dual-mode imaging and pH/NIR-responsive drug delivery. Nano-Micro Lett..

[24] Papa S, Vismara I, Mariani A, Barilani M, Rimondo S (2018). Mesenchymal stem cells encapsulated into biomimetic hydrogel scaffold gradually release CCl_2_ chemokine in situ preserving cytoarchitecture and promoting functional recovery in spinal cord injury. J. Control. Release.

[25] Caron I, Rossi F, Papa S, Aloe R, Sculco M (2016). A new three dimensional biomimetic hydrogel to deliver factors secreted by human mesenchymal stem cells in spinal cord injury. Biomaterials.

[26] Cerqueira SR, Oliveira JM, Silva NA, Leite-Almeida H, Ribeiro-Samy S (2016). Microglia response and in vivo therapeutic potential of methylprednisolone-loaded dendrimer nanoparticles in spinal cord injury. Small.

[27] Naderi N, Karponis D, Mosahebi A, Seifalian AM (2018). Nanoparticles in wound healing; from hope to promise, from promise to routine. Front. Biosci..

[28] Wang X, Li X, Ito A, Watanabe Y, Sogo Y, Tsuji NM, Ohno T (2016). Stimulation of in vivo antitumor immunity with hollow mesoporous silica nanospheres. Angew. Chem. Int. Ed..

[29] Song B, Wu C, Chang J (2012). Controllable delivery of hydrophilic and hydrophobic drugs from electrospun poly(lactic-co-glycolic acid)/mesoporous silica nanoparticles composite mats. J. Biomed. Mater. Res., Part B.

[30] Mekaru H, Lu J, Tamanoi F (2015). Development of mesoporous silica-based nanoparticles with controlled release capability for cancer therapy. Adv. Drug Del. Rev..

[31] Wang Y, Zhao Q, Han N, Bai L, Li J (2015). Mesoporous silica nanoparticles in drug delivery and biomedical applications. Nanomed. Nanotechnol. Biol..

[32] Bharti C, Nagaich U, Pal AK, Gulati N (2015). Mesoporous silica nanoparticles in target drug delivery system: a review. Int. J. Pharma. Investig..

[33] Mann AP, Scodeller P, Hussain S, Joo J, Kwon E (2016). A peptide for targeted, systemic delivery of imaging and therapeutic compounds into acute brain injuries. Nat. Commun..

[34] Wang Q, Zhang H, Xu H, Zhao Y, Li Z (2018). Novel multi-drug delivery hydrogel using scar-homing liposomes improves spinal cord injury repair. Theranostics.

[35] Zhang K, Xu L-L, Jiang J-G, Calin N, Lam K-F (2013). Facile large-scale synthesis of monodisperse mesoporous silica nanospheres with tunable pore structure. J. Am. Chem. Soc..

[36] He Q, Shi J, Chen F, Zhu M, Zhang L (2010). An anticancer drug delivery system based on surfactant-templated mesoporous silica nanoparticles. Biomaterials.

[37] Liu SJ, Liu SY (2015). Study on acute toxicity of arctigenin injection in SD rats. Chinese J. Pharmacovigilance.

[38] Ma SF, Chen Y-J, Zhang J-X, Shen L, Wang R, Zhou J-S, Hu J-G, Lu H-Z (2015). Adoptive transfer of M2 macrophages promotes locomotor recovery in adult rats after spinal cord injury. Brain Behav. Immun..

[39] Basso DM, Fisher LC, Anderson AJ, Jakeman LB, McTigue DM, Popovich PG (2006). Basso mouse scale for locomotion detects differences in recovery after spinal cord in injury in five common mouse strains. J. Neurotrauma.

[40] Rao J, Yang Y, Lin S, Shen J, Yan Y (2015). Repair of spinal cord injury by chitosan scaffold with glioma ECM and SB216763 implantation in adult rats. J. Biomed. Mater. Res., Part A.

[41] Waisman A, Hauptmann J, Regen T (2015). The role of IL-17 in CNS diseases. Acta Neuropathol..

[42] Assinck P, Duncan GJ, Hilton BJ, Plemel JR, Tetzlaff W (2017). Cell transplantation therapy for spinal cord injury. Nat. Neurosci..

[43] Pardridge WM (1999). Non-invasive drug delivery to the human brain using endogenous blood–brain barrier transport systems. Pharm. Sci. Technol. Today.

[44] Sun Q, Radosz M, Shen Y (2012). Challenges in design of translational nanocarriers. J. Control. Release.

[45] Saxena T, Loomis KH, Pai SB, Karumbaiah L, Gaupp E, Patil K, Patkar R, Bellamkonda RV (2015). Nanocarrier-mediated inhibition of macrophage migration inhibitory factor attenuates secondary injury after spinal cord injury. ACS Nano.

[46] Pillai DR, Dittmar MS, Baldaranov D, Heidemann RM, Henning EC, Schuierer G, Bogdahn U, Schlachetzki F (2009). Cerebral ischemia-reperfusion injury in rats-A 3 T MRI study on biphasic blood-brain barrier opening and the dynamics of edema formation. J. Cereb. Blood Flow Metab..

[47] Stewart PA, Farrell CR, Farrell CL, Hayakawa E (1992). Horseradish peroxidase retention and washout in blood–brain barrier lesions. J. Neurosci. Methods.

[48] Gaudin A, Yemisci M, Eroglu H, Lepetre-Mouelhi S, Turkoglu OF (2015). Erratum: Squalenoyl adenosine nanoparticles provide neuroprotection after stroke and spinal cord injury. Nat. Nanotechnol..

[49] Donnelly DJ, Popovich PG (2008). Inflammation and its role in neuroprotection, axonal regeneration and functional recovery after spinal cord injury. Exp. Neurol..

[50] Profyris C, Cheema SS, Zang DW, Azari MF, Boyle K, Petratos S (2004). Degenerative and regenerative mechanisms governing spinal cord injury. Neurobiol. Dis..

[51] Song J, Li N, Xia Y, Gao Z, Zou S-F (2016). Arctigenin confers neuroprotection against mechanical trauma injury in human neuroblastoma SH-SY5Y cells by regulating miRNA-16 and miRNA-199a expression to alleviate inflammation. J. Mol. Neurosci..

[52] Wu X, Yang Y, Dou Y, Ye J, Bian D (2014). Arctigenin but not arctiin acts as the major effective constituent of *Arctium lappa* L. Fruit for attenuating colonic inflammatory response induced by dextran sulfate sodium in mice. Int. Immunopharmacol..

[53] Shichita T, Sugiyama Y, Ooboshi H, Sugimori H, Nakagawa R (2009). Pivotal role of cerebral interleukin-17-producing gamma γδT cells in the delayed phase of ischemic brain injury. Nat. Med..

[54] Hill F, Kim CF, Gorrie CA, Moalem-Taylor G (2011). Interleukin-17 deficiency improves locomotor recovery and tissue sparing after spinal cord contusion injury in mice. Neurosci. Lett..

[55] Stammers AT, Liu J, Kwon BK (2012). Expression of inflammatory cytokines following acute spinal cord injury in a rodent model. J. Neurosci. Res..

[56] Kelley KW, Rowitch DH (2016). Astrocytes: the final frontier. Neuron.

[57] Pekny M, Pekna M, Messing A, Steinhaeuser C, Lee J-M (2016). Astrocytes: a central element in neurological diseases. Acta Neuropathol..

[58] Anderson MA, Burda JE, Ren Y, Ao Y, O’Shea TM (2016). Astrocyte scar formation aids central nervous system axon regeneration. Nature.

[59] Sofroniew MV (2015). Astrocyte barriers to neurotoxic inflammation. Nat. Rev. Neurosci..

[60] Hara M, Kobayakawa K, Ohkawa Y, Kumamaru H, Yokota K (2017). Interaction of reactive astrocytes with type I collagen induces astrocytic scar formation through the integrin-N-cadherin pathway after spinal cord injury. Nat. Med..

[61] Taylor PR, Roy S, Leal SM, Sun Y, Howell SJ, Cobb BA, Li X, Pearlman E (2014). Activation of neutrophils by autocrine IL-17A-IL-17RC interactions during fungal infection is regulated by IL-6, IL-23, RORγt and dectin-2. Nat. Immunol..

[62] Colombo E, Farina C (2016). Astrocytes: key regulators of neuroinflammation. Trends Immunol..

[63] Brambilla R, Morton PD, Ashbaugh JJ, Karmally S, Lambertsen KL, Bethea JR (2014). Astrocytes play a key role in eae pathophysiology by orchestrating in the CNS the inflammatory response of resident and peripheral immune cells and by suppressing remyelination. Glia.

