# Supplementary material for: Synthesis and Characterization of a Silica-Based Drug Delivery System for Spinal Cord Injury Therapy
Source: Nanomicro Lett. 2019 Mar 11;11:23. doi: 10.1007/s40820-019-0252-6 (PMC7770885; doi:10.1007/s40820-019-0252-6)
Supplement: Supplementary file 1 — Supplementary material 1 (PDF 292 kb) [file 40820_2019_252_MOESM1_ESM.pdf]

Supporting Information for

Synthesis and Characterization of a Silica-Based Drug Delivery System for Spinal Cord Injury Therapy

Guodong Sun[2], Shenghui Zeng[1], Xu Liu[1], Haishan Shi[1], Renwen Zhang[3], Baocheng Wang[4], Changren Zhou[1], Tao Yu[1,*]

[1]College of Chemistry and Material Sciences, Jinan University, Guangzhou 510632, People's Republic of China

[2]Department of Orthopedics, First Affiliated Hospital, Jinan University, Guangzhou 510632, People's Republic of China

[3]College of Traditional Chinese Medicine, Jinan University, Guangzhou 510632, People's Republic of China

[4]Tsinghua-Berkeley Shenzhen Institute, Tsinghua University, Shenzhen 518055, People's Republic of China

* Corresponding author. E-mail: ytbiom@jnu.edu.cn (Tao Yu)

S1 Experimental Section

S1.1 Preparation of Cluster-Like Mesoporous Silica (C-MSN) Powder

C-MSN was synthesized using a soft-templating method [29]. A mixture of 1.90 g of cetyl-trimethylammonium tosylate (CTATos, MERK) and 0.35 g of triethanolamine ($TEAH_3$) in 0.1 L of deionized water was stirred at 80 °C for 1 h and 14.58 g of tetraethyl-orthosilicate (TEOS) was quickly added into the surfactant solution. The mixture was stirred at 80 °C with a stirring speed of 1200 rpm for another 2 h. The synthesized C-MSNs were filtered, washed with water, and dried in the oven at 100 °C for 20 h.

S1.2 Preparation of Amino-Chemical C-MSN-NH_2

First, 7 mL anhydrous ethanol was added into 0.2 mL 3-aminopropyltriethoxysilane (APTES), which was dispersed for 1 h, named A solution. Next, 0.05 g MSN was added to 8 mL anhydrous ethanol to generate B solution. A and B solutions were mixed at 40 °C under water bath conditions for 8 h. After the reaction, the products were washed

three times with anhydrous ethanol, the samples were frozen and dried, and the amino-chemical C-MSN-NH_2 powder was obtained.

S1.3 Preparation of C-MSN-FITC (MSN-F) and C-MSN-FITC-CAQK (MSN-FC)

A total of 5 μL FITC or FITC-CAQK (Zhejiang Ontores Biotechnologies Co.), 0.0020 g 1-(3-Dimethylaminopropyl)-3-ethylcarbodiimide hydrochloride (EDC) and 0.0012 g N-Hydroxysuccinimide (NHS) were added to 20 mL deionized water. The solution was dispersed for 30 min, and the pH value of the solution was adjusted to 5.5 with 0.1 M HCl, yielding C solution. Then, 10 mg C-MSN-NH_2 was dispersed in 5 mL 0.1 M NaOH, named D solution, and the C solution was dropped slowly into D solution. Next, 0.1 M NaOH was used to adjust the pH of the solution to 6. After reacting at ambient temperature for 6 h, the product was obtained by centrifugation and washed three times with deionized water. MSN-F or MSN-FC were obtained after freeze-drying.

S1.4 Preparation of C-MSN/FITC-CAQK/ARC-G (MSN-FC@ARC-G)

A total of 10 mg MSN-FC was added to 0.1 mg mL^{-1} ARC-G solution and soaked at 4 °C for 24 h. The sample was obtained after centrifugal freeze drying.

S1.5 Characterization of MSNs

Characterization of MSNs: The particle analysis was measured on a Nano-ZS instrument (Malvern Instruments Limited); the surface chemical compositions of the dried samples were analyzed by X-ray photoelectron spectroscopy (XPS; Axis Ultra DLD, Kratos, Britain).

S1.6 Cell Culture

All cell-culture-related reagents were purchased from HyClone. RAW 264.7 cells (a mouse macrophage cell line, provided by Gao'lab of Jinan University, China) were cultured in RPMI-1640 medium supplemented with 10% fetal bovine serum (FBS) and 1% penicillin/streptomycin in a 5% CO_2-humidified chamber at 37 °C. After culturing for 12 h, the cell culture medium was replaced with 0.06 mg mL^{-1} of C-MSNs and after another 12 h for confocal imaging.

S2 Supplementary Results

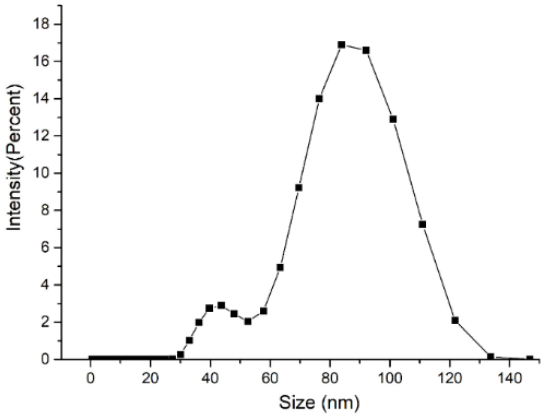

Fig. S1 The particle analysis of the C-MSNs

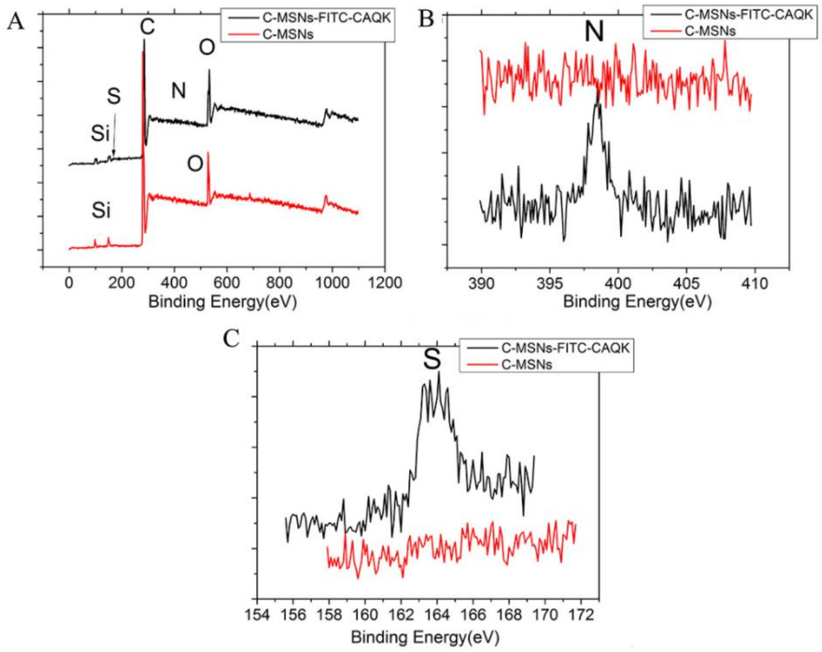

Fig. S2 A XPS full spectra for Nano system; **B** N 1s spectra; **C** S 2p spectra

The particle analysis of the C-MSNs (Fig. S1) showed that the particle size of C-MSNs was mainly distributed at 60-120 nm. X-ray photoelectron spectroscopy (XPS) measurements were used to further identify the surface analysis of the nano system (Fig. S2A, C). Characteristic peaks of silicon and oxygen were present in the spectrum of C-MSNs (Fig. S2A). The presence of S\N peaks in the XPS of MSN-FC@ARC-G demonstrated that MSNs were grafted by FITC-CAQK (Fig. S2A). In addition, the chemical interactions in MSN-FC@ARC-G were analyzed by the transitions in the N and S spectra and changes in the bonding energy (Fig. S2B, C). Based on these results, C-MSNs were successfully grafted by FITC-CAQK.

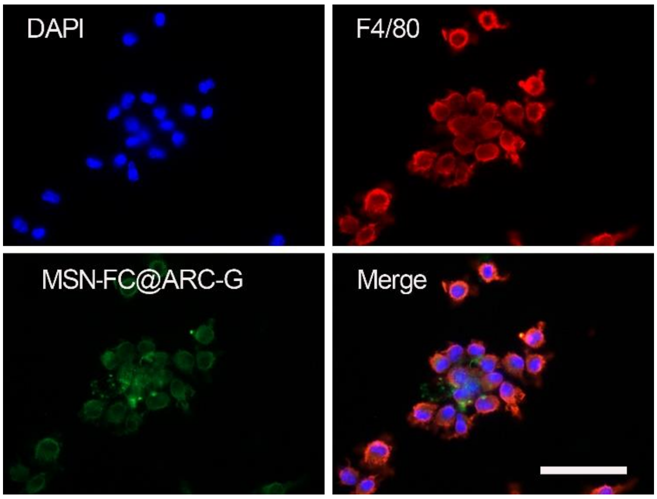

Fig. S3 Phagocytosis of MSN-ARC-G by macrophages (Scale bar: 50 μm)

To validate whether or not nanoparticles can interact with macrophages; Fluorescence-labeling nanoparticles were co-cultured with macrophages (Fig. S3), and the results show that almost all of nanoparticles were phagocytized by macrophages.

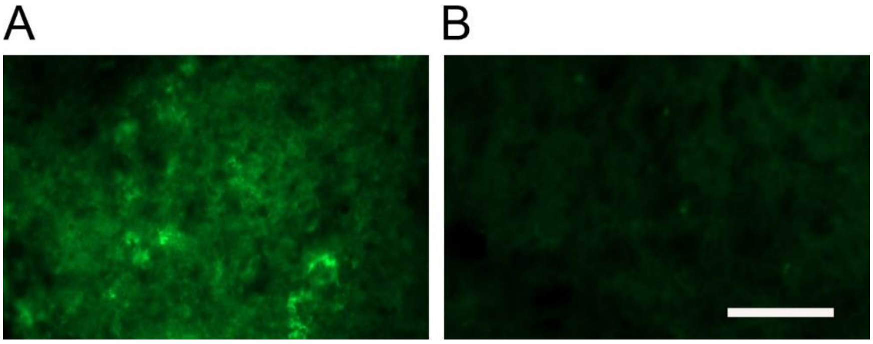

Fig. S4 MSN-FC@ARC-G penetrate the damaged blood spinal cord barrier. Fluorescent imaging of spinal cord **A** 24 h post-injury, **B** Sham operation (Scale bar: 50 μm)

To determine the permeability of MSN-FC@ARC-G to the injured spinal cord, we intravenously injected a (FITC)-labeled MSN-FC@ARC-G and fluorescent imaging at 24 h post-injury. It's observable that green fluorescence-representing MSN-FC@ARC-G extravasated into the injury spinal cord 24 h after injury; sham operation group can't be observed in the spinal cord tissue.
